# QOI4/361: Quantification of Inbound Links to Pediatric Web Sites as a Tool for Their Evaluation

**DOI:** 10.2196/jmir.1.suppl1.e97

**Published:** 1999-09-19

**Authors:** AA Hernández-Borges, ML Torres-Álvarez de Arcaya, P Macías-Cervi, MA Gaspar-Guardado, A Ruíz-Rabaza, R García-Closas

**Affiliations:** ^1^Hospital Universitario de Canarias, Canary Islands, La LagunaSpain

**Keywords:** Health Education, Information Systems, Computer Communication Networks, Internet, Bibliometrics, Webometrics

## Abstract

**Introduction:**

Some organizations have established a set of minimal standards every medical web site should meet. On the other hand, certain evaluation and rating systems rank plenty of web resources following subjective criteria. Finally, some authors have proposed the citation analysis of medical resources on the Web as a democratic way to evaluate and rank large amounts of medical web sites.

**Methods:**

A sample of pediatric web sites compiled from eight evaluation and rating systems which give the results of their evaluation as numeric or analog scales were studied in april 1998 about three characteristics: number of daily visits, time since their last update, and the number of inbound links. The reliability of those three characteristics as quality markers was studied by comparing them to the web sites evaluations, and the same web sites were studied again a year later in order to know their predicitive power as quality indicators. In addition, we studied some cybermetric parameters defined in basis to the number of inbound links and the size of each web site, which were calculated by means of the search engine Infoseek.

**Results:**

363 pediatric web sites were included in the 1998 survey. After removing some not available web sites (11%), we studied 134 domains or subdirectories and 188 web sites in 1999. The variables showed certain stability a year later, that is, the more updated and visited a web site was, and the higher number of inbound links received in 1998, the more updated, visited and linked was in 1999 (rS=0.69, p<0.001; rS=0.87, p<0.001; and rS=0.92, p<0.001, respectively). In the 1999 study, several quality markers were found. So, the number of inbound links, their absolute increment for the last year, the grade of web sites update, and the size (number of pages) of the domains and subdirectories, significantly correlated with their evaluations by the rating systems.

**Discussion:**

This study demonstrates not only that certain web sites characteristics are good quality markers of them, but also that they have certain predictive power as quality indicators. Similarly, a dinamic quality parameter has risen, such as the absolute increment of inbound links for a period of time. Certain cybermetric parameters based on the absolute number or the increment of inbound links to a medical web site could be good cybermetric indexes. However, this method have got some technical limitations. For instance, it is sensible to changes in the syntaxis of the web sites URLs, and it depends on the power of the search engine database we employe. In any case, a ranking based on this method would be so reliable as one based in the web sites evaluation by third parties. Moreover, some cybermetric indexes based on this method could provide a means of dinamic evaluation of a given web site

